# Identifying Higher-Volume Antibiotic Outpatient Prescribers Using Publicly Available Medicare Part D Data — United States, 2019

**DOI:** 10.15585/mmwr.mm7106a3

**Published:** 2022-02-11

**Authors:** Katryna A. Gouin, Katherine E. Fleming-Dutra, Sharon Tsay, Destani Bizune, Lauri A. Hicks, Sarah Kabbani

**Affiliations:** ^1^Chenega Corporation, Anchorage, Alaska; ^2^Division of Healthcare Quality Promotion, National Center for Emerging and Zoonotic Infectious Diseases, CDC.

Antibiotic prescribing can lead to adverse drug events and antibiotic resistance, which pose ongoing urgent public health threats (*1*). Adults aged ≥65 years (older adults) are recipients of the highest rates of outpatient antibiotic prescribing and are at increased risk for antibiotic-related adverse events, including *Clostridioides difficile* and antibiotic-resistant infections and related deaths (*1*). Variation in antibiotic prescribing quality is primarily driven by prescribing patterns of individual health care providers, independent of patients’ underlying comorbidities and diagnoses (*2*). Engaging higher-volume prescribers (the top 10% of prescribers by antibiotic volume) in antibiotic stewardship interventions, such as peer comparison audit and feedback in which health care providers receive data on their prescribing performance compared with that of other health care providers, has been effective in reducing antibiotic prescribing in outpatient settings and can be implemented on a large scale (*3*–*5**)*. This study analyzed data from the Centers for Medicare & Medicaid Services (CMS) Part D Prescriber Public Use Files (PUFs)* to describe higher-volume antibiotic prescribers in outpatient settings compared with lower-volume prescribers (the lower 90% of prescribers by antibiotic volume). Among the 59.4 million antibiotic prescriptions during 2019, 41% (24.4 million) were prescribed by the top 10% of prescribers (69,835). The antibiotic prescribing rate of these higher-volume prescribers (680 prescriptions per 1,000 beneficiaries) was 60% higher than that of lower-volume prescribers (426 prescriptions per 1,000 beneficiaries). Identifying health care providers responsible for a higher volume of antibiotic prescribing could provide a basis for additional assessment of appropriateness and outreach. Public health organizations and health care systems can use publicly available data to guide focused interventions to optimize antibiotic prescribing to limit the emergence of antibiotic resistance and improve patient outcomes.

Approximately 70% of Medicare beneficiaries are enrolled in Medicare Part D, the prescription drug benefit program for adults aged ≥65 years and persons with disabilities or end-stage renal disease. CMS Medicare Part D Prescribers by Provider is a publicly available data set that contains prescriber-level aggregate counts of outpatient prescription drug events by three drug types (antibiotics, antipsychotics, and opioids) and provider characteristics, including names, National Provider Identifier, specialty (including prescriber type), and zip code. There is a 2-year lag in data availability, during which prescription drug claims are finalized. Because beneficiary and antibiotic claim counts fewer than 11 are suppressed, the 2019 Medicare Part D Prescribers by Provider data set was used to assess prescriber-level antibiotic prescriptions among health care providers in the United States who distributed 11 or more antibiotic prescriptions.

Higher-volume prescribers were defined as those in the highest 10th percentile of prescriber-level antibiotic volume (number of antibiotic prescriptions filled) across all Medicare providers nationwide. The cumulative percentage of antibiotic volume prescribed by higher-volume prescribers was assessed overall, and the percentage of higher-volume prescribers in each U.S. Census Bureau region^†^ and specialty were described. To verify that antibiotic volume was not exclusively driven by the number of Medicare beneficiaries attributed to an individual prescriber, the percentage of beneficiaries with an antibiotic prescription and the prescriber’s antibiotic volume per 1,000 beneficiaries were calculated. The antibiotic prescribing rate was compared between the defined national subset of higher-volume prescribers and lower-volume prescribers by specialty and U.S. Census Bureau region. Ten beneficiaries were imputed for suppressed beneficiary counts to provide a conservative estimate of the prescribing rate. The Wilcoxon rank-sum test was used to compare median prescribing rates among prescribers. All analyses were performed using SAS (version 9.4; SAS Institute). This activity was reviewed by CDC and was conducted consistent with applicable federal law and CDC policy.^§^

During 2019, the Medicare Part D Prescribers by Provider data set included 1.2 million prescribers. After excluding prescribers with fewer than 11 antibiotic prescriptions and those in U.S. territories or overseas military bases, 697,065 (56%) prescribers were included in the analysis. A total of 59.4 million antibiotic prescriptions were filled by Part D beneficiaries, with a median of 47 (IQR = 23–100) antibiotic prescriptions per prescriber. Among all antibiotic prescriptions, 41% (24.4 million) were written by the top 10% (69,835) of antibiotic prescribers by number of prescriptions written (antibiotic volume) (Figure); these prescribers wrote a median of 284 antibiotic prescriptions (IQR = 230–393) compared with a median of 41 (IQR = 21–78) among lower-volume prescribers. Higher-volume prescribers prescribed antibiotics to a median of 38% of their patient panel (i.e., group of patients assigned to a specific health care provider or clinical team) compared with a median of 32% among lower-volume prescribers. In addition, the median antibiotic prescribing rate among higher-volume prescribers was 60% higher than that of lower-volume prescribers (680 versus 426 prescriptions per 1,000 beneficiaries) (p<0.001).

**FIGURE:**
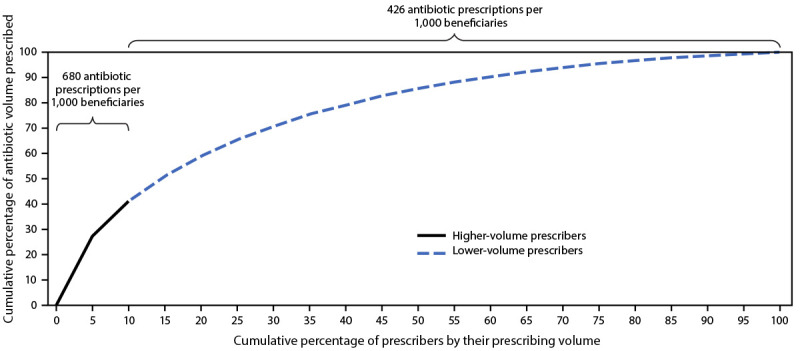
Cumulative percentage of antibiotics prescribed by Medicare Part D* prescribers, by prescribing volume and rate among higher-volume and lower-volume prescribers^†^ — United States, 2019 * Centers for Medicare & Medicaid Services Part D Prescribers by Provider data set, 2019. ^†^ Higher-volume prescribers are the top 10% of prescribers by antibiotic volume; lower-volume prescribers are the lower 90% of prescribers by antibiotic volume.

Approximately one half (48%) of higher-volume prescribers practiced in the South and prescribed 49% (12.3 million) of the total antibiotic prescriptions in this region (Table). Higher-volume prescribers in the South also had the highest median antibiotic prescribing rate (696 antibiotic prescriptions per 1,000 beneficiaries) compared with higher-volume prescribers in other regions (649 in the West) (p<0.001). The most common specialties of higher-volume prescribers were family practice and internal medicine, with 21% (19,213 of 89,759) and 20% (17,185 of 85,442) of prescribers, respectively classified as higher-volume prescribers. Family practice and internal medicine higher-volume prescribers accounted for approximately 60% of the antibiotics prescribed within their respective specialties and 22% of the total antibiotic volume, collectively. Although urologists only contributed 1% of the total prescriber number during 2019, one half (50%) of urologists were higher-volume prescribers and prescribed 2.0 million antibiotic prescriptions, or 83% of urology-prescribed antibiotic volume. Higher-volume prescribers, as expected, had higher antibiotic prescribing rates within each specialty, with the highest rate among dentists.

**TABLE:** Number of antibiotic prescribers, number of outpatient antibiotic prescriptions, and prescribing rate per 1,000 beneficiaries* among higher-volume prescribers and lower-volume prescribers,^†^ by U.S. Census Bureau region and specialty — United States, 2019

Characteristics	Higher-volume prescribers (top 10%)			Lower-volume prescribers (lower 90%)			Total prescribers^§^		
	Prescribers	Prescriptions	Prescriptions per 1,000 beneficiaries	Prescribers	Prescriptions	Prescriptions per 1,000 beneficiaries	Prescribers	Prescriptions	Prescriptions per 1,000 beneficiaries
	(n = 69,835)	(n = 24.4 million)		(n = 627,230)	(n = 35.0 million)		(N = 697,065)	(N = 59.4 million)	
	No. (%)	No. (%)	Median (IQR)	No. (%)	No. (%)	Median (IQR)	No. (%)	No. (%)	Median (IQR)
**U.S. Census Bureau region^¶^**									
South	33,571 (48.1)	12,277,664 (50.3)	696 (516–925)	217,854 (34.7)	12,800,940 (36.6)	434 (250–714)	251,425 (36.1)	25,078,604 (42.2)	471 (277–765)
Midwest	15,096 (21.6)	5,163,003 (21.2)	681 (507–912)	141,561 (22.6)	8,110,378 (23.2)	435 (260–714)	156,657 (22.5)	13,273,381 (22.4)	461 (278–750)
Northeast	11,188 (16.0)	3,715,665 (15.2)	655 (472–893)	129,416 (20.6)	6,802,148 (19.4)	410 (224–708)	140,604 (20.2)	10,517,813 (17.7)	432 (238–736)
West	9,980 (14.3)	3,241,995 (13.3)	649 (467–879)	138,399 (22.1)	7,270,835 (20.8)	419 (230–731)	148,379 (21.3)	10,512,830 (17.7)	436 (240–750)
**Specialty**									
Family practice	19,213 (27.5)	6,815,010 (27.9)	611 (463–796)	70,546 (11.2)	5,341,667 (15.3)	303 (201–455)	89,759 (12.9)	12,156,677 (20.5)	358 (225–553)
Internal medicine	17,185 (24.6)	6,476,428 (26.5)	590 (429–816)	68,257 (10.9)	4,716,606 (13.5)	333 (209–477)	85,442 (12.3)	11,193,034 (18.8)	375 (237–545)
Nurse practitioner	9,857 (14.1)	2,920,894 (12.0)	711 (553–866)	98,182 (15.7)	5,934,913 (17.0)	398 (244–587)	108,039 (15.5)	8,855,807 (14.9)	425 (258–625)
Urology	4,738 (6.8)	2,020,285 (8.3)	760 (603–961)	4,687 (0.7)	426,424 (1.2)	500 (370–660)	9,425 (1.4)	2,446,709 (4.1)	632 (462–839)
Physician assistant	5,200 (7.4)	1,553,698 (6.4)	686 (537–816)	61,273 (9.8)	3,634,949 (10.4)	407 (251–567)	66,473 (9.5)	5,188,647 (8.7)	427 (265–594)
Dentist	2,063 (3.0)	552,858 (2.3)	1,271 (1,122–1,450)	110,629 (17.6)	5,004,506 (14.3)	1,068 (914–1,222)	112,692 (16.2)	5,557,364 (9.4)	1,071 (917–1,228)
Other**	11,579 (16.6)	4,059,154 (16.6)	850 (583–1,239)	213,656 (34.1)	9,925,236 (28.4)	360 (188–533)	225,235 (32.3)	13,984,390 (23.5)	375 (197–560)

**Abbreviation**: CMS = Centers for Medicare & Medicaid Services.

* CMS Part D Prescribers by Provider data set, 2019.

^†^ Higher-volume prescribers are the top 10% of prescribers by antibiotic volume; lower-volume prescribers are the lower 90% of prescribers by antibiotic volume.

^§^ Total number of prescribers includes prescribers with ≥11 antibiotic prescription drug events filled at their direction by Medicare Part D beneficiaries during 2019.

^¶^ U.S. Census Bureau regions: *Northeast*: Connecticut, Maine, Massachusetts, New Hampshire, New Jersey, New York, Pennsylvania, Rhode Island, and Vermont. *Midwest*: Illinois, Indiana, Iowa, Kansas, Michigan, Minnesota, Missouri, Nebraska, North Dakota, Ohio, South Dakota, and Wisconsin. *South*: Alabama, Arkansas, Delaware, District of Columbia, Florida, Georgia, Kentucky, Louisiana, Maryland, Mississippi, North Carolina, Oklahoma, South Carolina, Tennessee, Texas, Virginia, and West Virginia. *West*: Alaska, Arizona, California, Colorado, Hawaii, Idaho, Montana, Nevada, New Mexico, Oregon, Utah, Washington, and Wyoming.

** “Other” includes the remaining provider specialties in the CMS Part D Prescribers by Provider data set. The top six prescriber specialties with the largest number of prescribers in the highest 10th percentile by antibiotic prescription volume are represented.

## Discussion

The goal of antibiotic stewardship is to improve the way health care providers prescribe antibiotics to optimize patient outcomes and reduce emergence of antibiotic resistance. During 2019, 41% of all Medicare Part D antibiotic prescriptions were prescribed by 10% of antibiotic prescribers, indicating that a small proportion of prescribers accounted for a disproportionately large number of antibiotic prescriptions. A similar 2016 study using claims data in Tennessee found that 50% of the state’s antibiotic volume was attributed to 9% of prescribers (*6*). This substantial difference in prescribing practices presents opportunities for improved prescribing through antibiotic stewardship activities focusing on these higher-volume prescribers, independent of specialty. Total antibiotic volume is associated with unnecessary prescribing rates and might be a reasonable proxy for unnecessary prescribing in primary care settings (*7*). Furthermore, higher-volume prescribers prescribed antibiotics to a larger share of their patient panel and their prescribing rate was 60% higher than that of lower-volume prescribers, indicating that their prescribing practices might be independent of the number of beneficiaries under their care. Thus, prioritizing higher-volume prescribers for focused stewardship interventions has the potential to have a sizeable impact on antibiotic prescribing, even when data on visit volume, prescribing indications, and appropriateness are not available.

This study demonstrates a way to identify antibiotic prescribers who account for a large proportion of prescribing and could provide a basis for additional assessment of appropriateness and outreach. For example, public health organizations could use Medicare Part D data to identify individual higher-volume antibiotic prescribers by specialty for focused stewardship interventions. The higher-volume prescribers in primary care specialties prescribed one-quarter of the total Medicare Part D antibiotic volume during 2019. Studies indicate that primary care providers have varying prescribing rates, suggesting opportunities for improvement in settings in which most antibiotics are prescribed (*5*). Urologists and dentists also have high prescribing rates and should be considered for antibiotic stewardship interventions (*8*,*9*). Further evaluation of prescribing practices by and within specialties and specific conditions are needed to identify areas for improvement in antibiotic prescribing. Similar to this analysis, studies have described higher rates of total outpatient antibiotic prescribing in the South (*8*), which could not be explained by differences in underlying conditions in older adults (*10*). Further evaluation of inequities in social determinants of health, underlying patient comorbidities, and access to care is needed to assess whether these factors might contribute to higher rates of prescribing observed in the South.

The publicly available CMS Part D Prescribers by Provider data set might enable public health organizations and health care systems to efficiently identify prescribers for stewardship outreach in their jurisdictions without the need for complex analytic methods or need to acquire prescription claims data or diagnosis data.^¶^ Prioritizing higher-volume prescribers for antibiotic stewardship interventions could facilitate larger reductions than targeting lower-volume prescribers. Prescriber feedback letters with peer comparison, which is an evidence-based, low-cost, and scalable intervention (*3*–*5*) can be used to engage specific health care providers or geographic areas.** In a randomized clinical trial among primary care physicians in Ontario, Canada receipt of a single letter informing prescribers they were in the top 25th percentile of prescribed antibiotic volume compared with their peers, along with recommendations about prescribing duration, resulted in a 5% relative reduction in total antibiotic use (*4*).

The findings in this report are subject to at least four limitations. First, the CMS Part D Prescribers by Provider data set captured prescription claims submitted to Medicare Part D and is thus not representative of the entire older adult population. Second, these data might not reflect health care providers’ prescribing behavior for their entire patient population and might overrepresent health care providers with a larger share of Medicare beneficiaries, patients with complex medical conditions, or visits for conditions for which antibiotics are prescribed. Third, this data only describes volume of prescribing and does not report diagnosis and underlying conditions; therefore, the data cannot be used to assess appropriateness of prescribing. Finally, the 2-year lag in data availability affects timeliness, which would be important for real-time audit and feedback. Nonetheless, these data are useful for characterizing provider prescribing behaviors and supporting public health stewardship outreach.

This report demonstrates how publicly available data might be leveraged to monitor antibiotic use and identify higher-volume prescribers. CMS Part D Prescribers by Provider data can be used by public health organizations and health care systems to guide antibiotic stewardship interventions and optimize antibiotic prescribing to limit the emergence of antibiotic resistance and improve patient outcomes.

**Summary**

**What is already known about this topic?**

Health care providers vary in their propensity to prescribe antibiotics. Peer comparison audit and feedback is an effective antibiotic stewardship intervention to improve antibiotic prescribing.

**What is added by this report?**

The highest 10% of antibiotic prescribers prescribed 41% of total antibiotic prescriptions for Medicare Part D beneficiaries in 2019. The antibiotic prescribing rate of these higher-volume prescribers was 60% higher than that of lower-volume prescribers.

**What are the implications for public health practice?**

Publicly available Medicare Part D data can be used by public health organizations and health care systems to guide antibiotic stewardship interventions and optimize antibiotic prescribing to limit the emergence of antibiotic resistance and improve patient outcomes.

## Footnotes

* https://data.cms.gov/provider-summary-by-type-of-service/medicare-part-d-prescribers/medicare-part-d-prescribers-by-provider (Accessed October 18, 2021).

† U.S. Census Bureau regions: *Northeast*: Connecticut, Maine, Massachusetts, New Hampshire, New Jersey, New York, Pennsylvania, Rhode Island, and Vermont. *Midwest*: Illinois, Indiana, Iowa, Kansas, Michigan, Minnesota, Missouri, Nebraska, North Dakota, Ohio, South Dakota, and Wisconsin. *South*: Alabama, Arkansas, Delaware, District of Columbia, Florida, Georgia, Kentucky, Louisiana, Maryland, Mississippi, North Carolina, Oklahoma, South Carolina, Tennessee, Texas, Virginia, and West Virginia. *West*: Alaska, Arizona, California, Colorado, Hawaii, Idaho, Montana, Nevada, New Mexico, Oregon, Utah, Washington, and Wyoming.

§ 45 C.F.R. part 46.102(l)(2), 21 C.F.R. part 56; 42 U.S.C. Sect. 241(d); 5 U.S.C. Sect. 552a; 44 U.S.C. Sect. 3501 et seq.

¶
https://www.cdc.gov/antibiotic-use/pdfs/Outpatient-Rx-Analytic-Guide-508.pdf

** CDC provides outpatient antibiotic stewardship resources, including prescriber feedback letters. https://www.cdc.gov/antibiotic-use/core-elements/outpatient.html
